# A Study on the Use of the Phyto-Courier Technology in Tobacco Leaves Infected by *Agrobacterium tumefaciens*

**DOI:** 10.3390/ijms241814153

**Published:** 2023-09-15

**Authors:** Annelie Gutsch, Roberto Berni, Jean-Francois Hausman, Flavia Maria Sutera, Ashkan Dehsorkhi, Nissim Torabi-Pour, Suzanne Saffie-Siebert, Gea Guerriero

**Affiliations:** 1 Environmental Research and Innovation Department, Luxembourg Institute of Science and Technology, 5, Rue Bommel, L-4940 Hautcharage, Luxembourg; annelie.gutsch@googlemail.com (A.G.); roberto.berni@list.lu (R.B.); jean-francois.hausman@list.lu (J.-F.H.); 2 SiSaf Ltd., Surrey Research Park, Guildford GU2 7RE, UK; sutera@sisaf.com (F.M.S.); dehsorkhi@sisaf.com (A.D.); torabipour@sisaf.com (N.T.-P.)

**Keywords:** silicon-stabilised hybrid lipid nanoparticles (sshLNPs), *Nicotiana benthamiana*, biotic stress, *Agrobacterium tumefaciens*, gene expression analysis, microscopy, quercetin

## Abstract

Climate change results in exceptional environmental conditions and drives the migration of pathogens to which local plants are not adapted. Biotic stress disrupts plants’ metabolism, fitness, and performance, ultimately impacting their productivity. It is therefore necessary to develop strategies for improving plant resistance by promoting stress responsiveness and resilience in an environmentally friendly and sustainable way. The aim of this study was to investigate whether priming tobacco plants with a formulation containing silicon-stabilised hybrid lipid nanoparticles functionalised with quercetin (referred to as GS3 phyto-courier) can protect against biotic stress triggered by *Agrobacterium tumefaciens* leaf infiltration. Tobacco leaves were primed via infiltration or spraying with the GS3 phyto-courier, as well as with a buffer (B) and free quercetin (Q) solution serving as controls prior to the biotic stress. Leaves were then sampled four days after bacterial infiltration for gene expression analysis and microscopy. The investigated genes increased in expression after stress, both in leaves treated with the phyto-courier and control solutions. A trend towards lower values was observed in the presence of the GS3 phyto-courier for genes encoding chitinases and pathogenesis-related proteins. Agroinfiltrated leaves sprayed with GS3 confirmed the significant lower expression of the pathogenesis-related gene *PR-1a* and showed higher expression of peroxidase and serine threonine kinase. Microscopy revealed swelling of the chloroplasts in the parenchyma of stressed leaves treated with B; however, GS3 preserved the chloroplasts’ mean area under stress. Furthermore, the UV spectrum of free Q solution and of quercetin freshly extracted from GS3 revealed a different spectral signature with higher values of maximum absorbance (A_max_) of the flavonoid in the latter, suggesting that the silicon-stabilised hybrid lipid nanoparticles protect quercetin against oxidative degradation.

## 1. Introduction

Climate change results in exceptional environmental conditions, such as increased heavy rainfalls or long, dry periods without any precipitation. These conditions favour pathogen infestation of plants, which induces biotic stress. On top, climate change drives the migration of pathogens [1,2] confronting local plants to a new and unknown stress, to which they are not yet adapted. Biotic stress disrupts plant metabolism and hampers plant fitness and performance with detrimental consequences on their productivity [3,4,5,6]. Plants have established mechanisms to cope with such stressful events in a fast and efficient way to eventually adapt and survive [7]. Tackling biotic stress is, however, physiologically costly and draws energy from biomass production, seeds, and, therefore, plant yield. To sustain productivity, it is vital to understand the molecular processes underlying stress response and to find possibilities to improve plant resistance by stimulating stress response signalling, for example, via the exogenous application of biostimulants in an environmentally friendly and sustainable way. 

Nanotechnology has become an important tool for sustainable agriculture [8,9]. Nanoparticles (NPs) can be used to alleviate stress symptoms in plants subjected to exogenous constraints, utilised to enhance plant nutrition and protection or to deliver a bio-active compound effectively into plant cells [10,11]. In that context, it was previously reported that the formulation based on silicon (Si)-stabilised hybrid lipid nanoparticles (sshLNPs) functionalised with quercetin (referred to as phyto-courier) is a promising technology to alleviate the negative effects of stresses in plants [12,13]. Quercetin is a secondary plant metabolite that belongs to the group of flavonoids and is synthesised via the phenylpropanoid pathway. It has known stress-mitigating properties and acts as an antioxidant, which protects plants against biotic and abiotic stressors [14,15,16,17,18]. Additionally, the phyto-courier releases orthosilisic acid Si(OH)_4_ over time, which is the form of Si that can be absorbed by plants and translocated to the aerial tissue where it is deposited as amorphous silica SiO_2_. The element Si contributes to reinforcing plant defence against (a)biotic stress via different mechanisms, the most evident being the structural protection by impregnating cell walls with SiO_2_ [19,20,21]. 

Si- and mesoporous silica-NPs (MSNs) themselves can have a direct impact on plants by affecting their physiology in multiple ways, such as increased protein abundance, synthesis of phenolic compounds, photosynthetic activity, and chlorophyll accumulation, which improve both plant growth and yield [22,23,24]. For example, a study on maize plants revealed that a correlation existed between silica NPs’ exposure and uptake vs. the response to stress. Further observations confirmed that soil amendment using silica NPs was even more effective than foliar treatment for maize plants [21], therefore suggesting the use of Si-NPs as fertiliser for sustainable agriculture. In wheat leaves treated with MSNs at 500, 1000, and 2000 mg L^−1^, an increase higher than 30% was observed in the content of chlorophyll *a*, while proteins increased by more than 17% [23]. A study performed on *Zea mays* underlined the beneficial effects of nanosilica on both enhancement of seed germination and chlorophyll content [25]. When investigated in *Glycine max*, the effect promoted by nanosilica was to reduce salt stress by boosting the antioxidant activities, metabolic processes increasing K^+^ and decreasing Na^+^ intracellular concentration, plus decreasing lipid peroxidation and reactive oxygen species (ROS) generation [26]. Furthermore, in salt-stressed textile hemp, sshLNPs alone mitigated the stress symptoms in the leaves which appeared more turgid when compared to control ones treated with buffer; additionally, under salinity, stress-related genes were expressed at lower levels in leaves sprayed with sshLNPs [12]. It was further speculated that Si-NPs add a structural layer to plant cells by strengthening the cell wall network, thus preventing/limiting the infection by various pests. Due to the porous surface of sshLNPs, they can be functionalised with bioactive compounds of choice and, as such, can serve to protect crops through the delivery of different molecules, for example, nucleic acids, proteins, or chemicals [27]. 

Under heavy metal stress, Si application promoted the abundance of proteins involved in cell wall differentiation [28]. Root-applied Si led to resistance against powdery mildew by activating defence-related enzymes in leaves [29]. This stimulatory effect makes Si an attractive candidate to support stress tolerance in plants, and its use in NPs-based formulations has two advantages: (1) elemental Si is released to support plant stress response and (2) the NPs act as delivery vehicles. Such a dual effect of Si-NPs was already proven to efficiently induce systemic acquired resistance against a biotic stressor [30]. 

Previously, publications on textile hemp [12] and tomato [13] focused on evaluating the ameliorative effects of quercetin-loaded sshLNPs in plants subjected to salinity. Here, the effect of the GS3 phyto-courier functionalised with 25 mg of quercetin was investigated in tobacco plants subjected to a biotic stress. Several studies demonstrated that Si is able to predispose the defence response in plants (priming), which is fully unfolded with the onset of the stress [19]. Leaves of *Nicotiana benthamiana* (tobacco) were infiltrated with *Agrobacterium tumefaciens* to provoke a biotic stress after having primed them, via injection or spraying, with either the phyto-courier or control solutions (consisting of buffer alone-B or free quercetin-Q). Spraying was here investigated as this application modality is easier than leaf infiltration in the perspective of an open field use.

*N. benthamiana* is a useful system to investigate the function of genes by transient expression through agroinfiltration or to study gene expression profiles under defined environmental conditions [31]. It is thus widely used in research to address various biological questions [32,33,34]. The advantage of agroinfiltration is that the experimental results can be generated within a few days. Combined with gene expression analysis via quantitative real-time PCR (qRT-PCR), it is an ideal method to investigate which genes are involved during stress response and how this response is altered when primed with the phyto-courier. The leaves primed with the formulations via spraying were also subjected to microscopy analysis. To understand if any differences were present in the stability of quercetin complexed with sshLNPs in the GS3 phyto-courier or the free form (Q solution), a qualitative assessment was performed by comparing the UV spectra of free quercetin and the flavonoid extracted from the sshLNPs. 

## 2. Results

### 2.1. Agroinfiltration Stimulated Stress-Related Genes, but Priming with GS3 Induced Some Significant Changes

Changes in gene expression caused by agroinfiltration were compared in leaves primed with the GS3 phyto-courier and B/Q control solutions. The different formulations were either injected into tobacco leaves with a needleless syringe or sprayed.

It must be noted that the variation of gene expression among biological replicates and treatments was very high in the leaves primed via injection, due to the mechanical stress caused by the forced entry of the viscous formulations containing hypromellose in the leaf parenchyma. Notwithstanding the variability, the Principal Component Analysis of the gene expression data revealed the presence of two separate groups corresponding to the control and *Agrobacterium*-infected samples (Figure S1a). Therefore, despite the mechanical stress caused during the priming phase, biotic stress responses could be discerned.

*Agrobacterium* provoked a strong increase in the expression values of stress-related genes (Figure S1b): the chitinases *Chit6*, *Chn*, *PR-Q*, the genes *VAS*, *PDRP1*, *PR-1a*, and *WIN* which code for lipid transfer, pleiotropic drug resistance, pathogenesis-related and wound-induced proteins, as well as the endo-1,3-beta-glucosidase *GEβGluc* were all induced in the agroinfiltrated leaves. No statistically significant differences could be observed when comparing the GS3-primed leaves with respect to B- and Q-treated ones. Only a trend towards lower values was observed in the presence of the phyto-courier (dotted boxes in Figure S1b).

Since the pathogenesis-related proteins showed a trend towards decreased expression, the experiment was repeated on leaves that were primed with the formulations via spraying prior to biotic stress. The objective was to verify whether statistically significant changes could be observed on samples that were not mechanically injured by priming. During this second experiment, genes involved in photosynthesis and known to be downregulated by agroinfiltration [34] were included in the analysis. These genes encode the oxygen-evolving enhancer protein 1 (OEE-1), the RuBisCO large subunit-binding protein subunit beta (RuBisCO-BP), a serine/threonine protein kinase (SerThrKin), and the photosystem I reaction centre subunit N (PSI-N).

**Figure 1:**
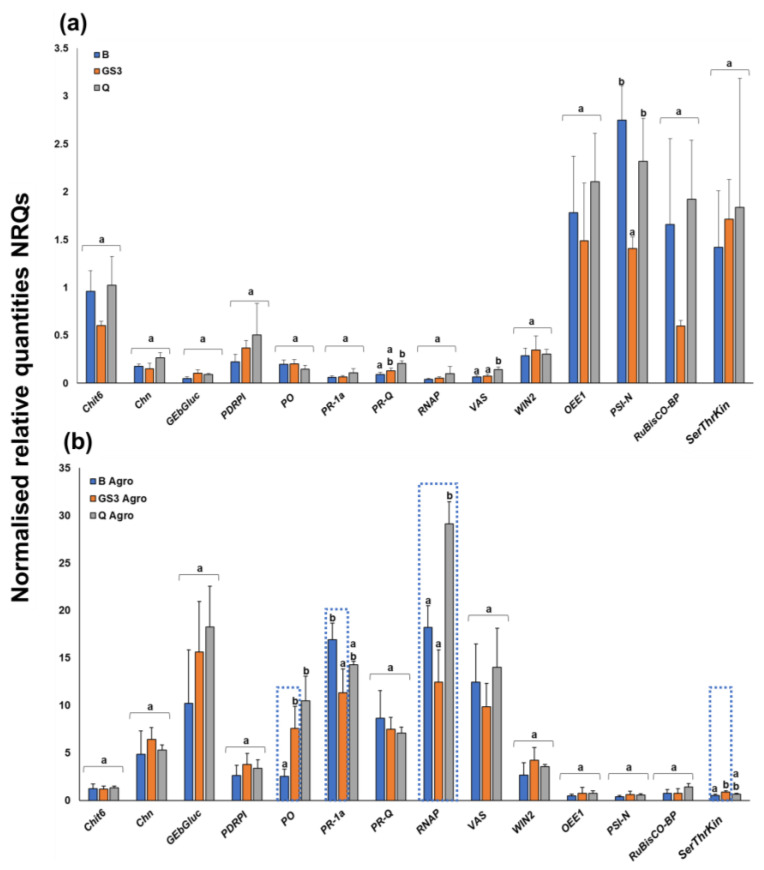
Gene expression data expressed as normalised relative quantities (NRQs); (**a**) control samples and (**b**) agroinfiltrated samples. The error bars refer to the standard deviation calculated from three biological replicates. Different letters indicate statistically significant differences among groups (*p*-value < 0.05). The statistical parameters are indicated in Table S1. The dotted areas show the different expression values in the GS3- or Q-treated leaves as compared to B-treated samples. B: buffer; GS3: phyto-courier formulation containing 25 mg of quercetin; Q: quercetin; Agro: agroinfiltrated.

Under control conditions, spraying of the Q formulation triggered a statistically significant increase in the expression of *PR-Q* and *VAS*, while GS3 significantly decreased *PSI-N* (Figure 1a). Upon biotic stress, *PR-1a* decreased significantly in leaves primed with GS3, while *PO* and *RNAP* increased significantly (Figure 1b). Q spraying also caused a significant increase in *PO* expression after agroinfiltration (Figure 1b).

### 2.2. Agroinfiltration Increased the Chloroplasts’ Mean Area and GS3 Prevented This Effect

**Figure 2:**
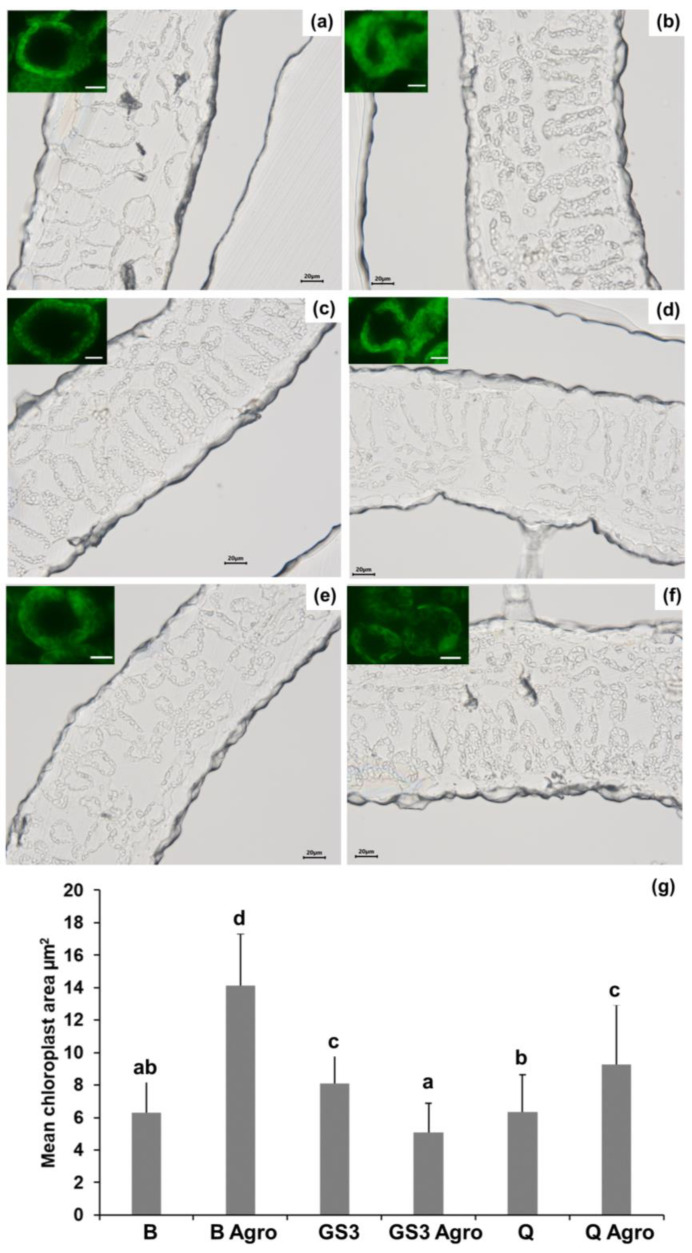
Optical microscopy of the leaf parenchyma in control and stressed leaves; (**a**) buffer-primed, (**b**) buffer-primed and agroinfiltrated, (**c**) GS3-primed, (**d**) GS3-primed and agroinfiltrated, (**e**) Q-primed, (**f**) Q-primed and agroinfiltrated, (**g**) plot of the mean chloroplast area calculated on *n* = 40 with ImageJ. Different letters indicate statistically significant differences among groups (*p*-value < 0.05) determined with a Kruskal–Wallis test with Dunn’s post hoc test (*X^2^*(5) = 124.765, *p*-value = 0.000). Insets in (**a**–**f**): chloroplast autofluorescence (bar = 10 µm).

To identify any histological changes triggered by the biotic stressor in tobacco leaves, optical microscopy was performed. Leaf cross sections (10 µm) were thus prepared at the microtome and observed. As can be seen in Figure 2, the palisade and spongy parenchyma of the leaves infiltrated with *Agrobacterium* and previously sprayed with B showed bigger chloroplasts compared to B-sprayed leaves without stress (Figure 2a,b). Treatment of the leaves with GS3 slightly, but significantly, increased the mean area under control conditions (Figure 2c), and under stress it prevented the swelling observed with B (Figure 2d). Q application could also reduce the swelling upon stress (Figure 2e,f), but the mean area was significantly higher than B under control conditions and GS3 under biotic stress (Figure 2g). 

In Figure 2g, the mean area of the chloroplasts in the palisade and spongy parenchyma is plotted, and the results confirm the microscopy observations: under stress, chloroplasts display a bigger mean area compared to control conditions, and GS3 spraying mitigates the swelling in a significant manner under biotic stress with values in the same range as control conditions (Figure 2g). 

### 2.3. Relative Flavonoid Accumulation and Antioxidant Power of the Leaf Metabolite Extracts, Phyto-Courier, and Control Solutions

As a following step, the relative flavonoid accumulation and the antioxidant capacity of extracts from tobacco leaves were measured. Flavonoids, such as quercetin, are antioxidants and accumulate during plant stress exposure in order to protect the plant [35]. They show a peak of absorbance at ≈330 nm in a full spectral analysis, which can be used to determine their relative accumulation [36]. 

**Figure 3:**
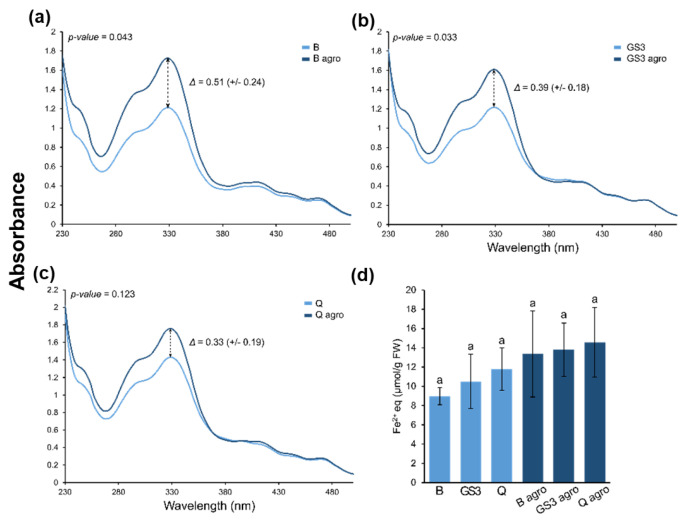
UV spectra (**a**–**c**) and antioxidant capacity (**d**) of metabolite extracts from tobacco leaves. Leaves were primed with the phyto-courier or control solutions prior to agroinfiltration. The relative accumulation of flavonoids was determined by considering the peak at 329 nm (**a**–**c**). Average antioxidant capacity from four replicates including standard deviation is expressed as µmol of Fe^2+^ equivalents (eq) per g of leaf sample. Letters indicate significance at *p*-value < 0.05 determined with an ANOVA one-way analysis followed by Tukey’s post hoc test (F(5) = 2.017, *p*-value = 0.125).

Agroinfiltration significantly increased the relative accumulation of flavonoids regardless of priming with GS3, B, or Q (Figure 3a–c). The highest peak at 329 nm and, therefore, the highest relative abundance of flavonoids was detected in metabolite extracts of agroinfiltrated samples primed with B and Q (Figure 3a,c), whereby the highest difference (Δ) in flavonoid accumulation between nonagroinfiltrated and agroinfiltrated samples was observed when samples were primed with B (Figure 3a). It must be noted that samples primed with Q displayed already a higher abundance of flavonoids under control conditions, and the increase in flavonoid abundance after agroinfiltration was limited, resulting in a lower Δ (Figure 3c) compared to samples primed with B and GS3 (Figure 3a and b, respectively). This was expected since free Q was injected in the leaf parenchyma which may have masked any stress-induced flavonoid accumulation.

In accordance with the UV spectra, the antioxidant capacity (expressed as µmol of Fe^2+^ equivalents per g of leaf sample) was by trend higher in agroinfiltrated samples (Figure 3d). However, injection of either the phyto-courier or control solutions had no statistically significant impact but showed a trend to higher antioxidant capacity after priming with GS3 and Q in nonagroinfiltrated as well as agroinfiltrated leaves primed with Q.

**Figure 4:**
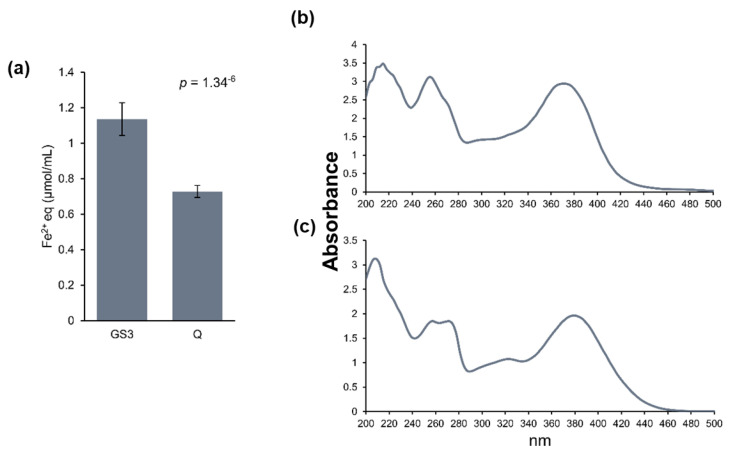
Qualitative evaluation of the GS3 phyto-courier and free Q solution. Antioxidant capacity determined by the FRAP assay. The antioxidant capacity is expressed as µmol of Fe^2+^ equivalents (equivalents, eq) per mL of solution. A *t*-test was performed to determine the significance with *p*-value < 0.05 (**a**). UV spectra of the quercetin extracted from the phyto-courier (**b**) and of the free Q solution (**c**). Displayed absorption values were reduced by absorption values of the solubilisation buffer alone (hypromellose/Pluronic L-61 and PBS buffer 1:1 *v*/*v*).

A FRAP assay was performed with the pure solutions used to prime tobacco leaves to gain general information about their antioxidant capacity. The solution of the GS3 phyto-courier had a significantly higher antioxidant capacity than the Q solution with free quercetin (Figure 4a). The antioxidant capacity of the buffer alone was “0” and is not shown. Additionally, any UV spectral differences of quercetin were assessed by extracting the flavonoid from the GS3 phyto-courier and comparing its UV spectrum with that of the free quercetin in the Q solution. The purpose was to evaluate, qualitatively, structural differences between the flavonoid freshly extracted from the sshLNPs and the free form. The spectral changes of quercetin extracted from the sshLNPs and of the free quercetin in the Q solution are shown in Figure 4b, and c, respectively. Quercetin characteristically shows two main peaks, one around 250 nm and a second around 380 nm [37]. Both peaks were clearly visible and sharp in the UV spectrum of the quercetin extracted from GS3 (Figure 4b). The spectral signature of free quercetin, despite showing the occurrence of the two peaks, also indicated a decrease in the A_max_ values of the two major bands (Figure 4c). This result demonstrates, although in a qualitative manner, that free quercetin is structurally different from the flavonoid freshly extracted from the sshLNPs. It should also be noted that the spectral signature of free quercetin revealed a shoulder around 330 nm which is absent in the flavonoid extracted from the sshLNPs. 

## 3. Discussion

The present study aimed to investigate whether priming a plant with the phyto-courier functionalised with 25 mg of quercetin protected against a biotic stress, here caused by *Agrobacterium*. Priming is a phenomenon whereby the plant reaches a physiological state that allows it to respond faster and more strongly to an exogenous stress [38,39]. In the primed state, the defences are pre-alerted through a mechanism that improves the perception and subsequent signalling, usually with minimal or no changes in gene expression [38]. 

Si is a quasi-essential metalloid that, when supplied to plants, allows them to grow more vigorously and enhances responsiveness to (a)biotic stresses [40]. Among the protective effects of Si, there is the formation of a protective layer of amorphous SiO_2_ which encrusts the cell walls, thereby physically hindering the penetration of pathogens [20,21]. Silicified structures have indeed been observed and reported in plants, from high accumulators (such as rice, horsetail) to nonaccumulators (such as hemp), and these include bulliform and silica cells, stomata, trichomes, as well as conductive tissues, namely, xylem vessels [41]. 

Regarding Si-NPs, it was reported in literature that pre-exposure to the NPs affected the response to a following stress. For example, wheat seed primed with Si-NPs reached a higher biomass and yield when exposed to Cd [42], and increased salt stress tolerance in *Lathyrus* seedlings was achieved [43]. In *Mellissa officinalis* L. plants, seed priming increased the abundance of primary and secondary metabolites when in combination with rhizobacteria inoculation [44]. However, most research on priming was undertaken in seeds, while here mature leaves of tobacco plants were primed with the phyto-courier through infiltration or spraying. 

In a previous study, the phyto-courier functionalised with quercetin (2.5 or 25 mg) was also sprayed several times on the leaves of plants (textile hemp and tomato) and shown to protect against salinity by acting as a nano-biostimulant [12,13]. Once injected or sprayed, quercetin is delivered into the leaf parenchyma, where it is progressively released, increasing its intracellular abundance before stress occurs, thus acting as a biostimulant. Natural compounds have been used as priming agents with superior results over synthetic counterparts [38], and among the most effective ones there are redox-active compounds such as thiamine [45], riboflavin [46], and quercetin [15,16,17,18,47].

Agroinfiltration is a fast and easy method to address fundamental biological questions, e.g., the functional role of genes such as transcription factors. The injection of agrobacteria into the leaf parenchyma induces transcriptional changes in genes of the plant immune response [34], and this response was here used to investigate whether priming with the phyto-courier would offer any mitigation in the induction of stress-responsive genes. Changes in gene expression caused by agroinfiltration in tobacco leaves were compared with those caused by infiltration of the buffer alone after priming with the phyto-courier. Agroinfiltration induced a strong upregulation of almost all genes of interest (Figure S1b), which outweighed any changes in the primed leaves. This is not surprising considering the strong mechanical and biotic stress to which the tobacco leaves were subjected during the experiment. Nevertheless, although statistics did not reveal significant changes, trends could be observed in the gene expression pattern in leaves primed with either the GS3 phyto-courier or the control solutions B and Q. The stress-related genes *Chit6*, *Chn*, *PR-Q*, as well as the genes *VAS*, *PDRP1*, *PR1a*, and *WIN* coding for lipid transfer, pleiotropic drug resistance, pathogenesis-related and wound-induced proteins, were expressed at lower levels compared to B and Q solutions (Figure S1b). The same behaviour was observed for the endo-1,3-beta-glucosidase *GEβGluc*. 

*Chn*, *GeβGluc*, and *WIN2* are specifically involved in plant defence [48,49,50]. Chitinases participate in the plant defence by breaking down chitin, a polysaccharide abundant in plant pathogens, but they seem to play an unspecific role during heavy metal stress [51]. Likewise, GEβGluc degrades fungal cell wall polysaccharides by hydrolysis and plays a role in the defence against pathogen attacks [52,53]. *WIN2* encodes a chitin-binding protein [54], and its transcription is induced by wounding and pathogens. Among the genes showing a trend towards lower expression in the presence of *Agrobacterium*, there is *PR-1a*, a member of the PR-1 family which is known to respond to pathogen attack [55]. The expression of this gene showed a statistically significant reduction under biotic stress after spraying with GS3 (Figure 1b), a finding which suggests a protective effect of the phyto-courier. The peroxidase *PO* increased in expression under stress after spraying with GS3 or Q formulations (Figure 1b). This peroxidase corresponds to the N1 isoform that is rapidly induced upon wounding to put in place a self-defence mechanism [56]. Interestingly, the regulation of the *PO* and *PR1* gene expression is different, as *PO* was shown to be suppressed by methyl jasmonate and coronatine, differently from *PR1* [56]. The *PO* expression pattern was also reported to be temporally distinct from *PR1*, since the induction of the latter was shown to be delayed with respect to *PO* and to maintain high levels for a longer time [56]. The distinct transcriptional regulation of these two genes can explain their different behaviour under biotic stress in response to GS3 priming. 

As expected, a statistically significant reduction in the expression of genes related to photosynthesis (*OEE1*, *RuBisCO-BP*, *SerThrKin*, and *PSI-N*) was observed after biotic stress (Figure 1); however, of these genes, the *SerThrKin* gene increased significantly in expression in GS3-sprayed leaves under stress (Figure 1b). This kinase is annotated as a homolog of *stt7*, which is involved in state transition and, more specifically, in LHCII phosphorylation and formation of a PSI–LHCI–LHCII supercomplex in *Chlamydomonas reinhardtii* [57]. This is an adaptive mechanism that balances the excitation of the two photosystems under varying light regimes [58,59]. Biotic stress is known to affect photosynthesis [60], and the increased expression of the kinase may represent an adaptive mechanism, under biotic stress, triggered at the chloroplast level after GS3 priming. It remains to be verified whether this mechanism is connected with the significant decrease in *PSI-N* expression under control conditions after GS3 treatment (Figure 1a).

Biotic stress caused by the infiltration in tobacco leaves of the strain here used, *Agrobacterium* GV3101 (pMP90), was shown to affect the chloroplasts’ position with respect to the nucleus and shape because of an increase in stromules and to impact starch content due to cytokinin secretion by the strain [61]. Optical microscopy in this study revealed that the chloroplasts’ size alterations were significantly reduced by GS3 under stress (Figure 2d,g), which could preserve the mean area of chloroplasts under control conditions (Figure 2a,g). Under control conditions, GS3 induced a small, but significant, increase in the mean area of the chloroplasts (Figure 2c,g): this phenomenon and its eventual relationship with a chloroplast priming state triggered by GS3 awaits further investigation. To summarise, the microscopic analyses confirmed the absence of stress-induced chloroplast alterations after treatment with the GS3 formulation.

Biotic stress induces oxidative stress via the generation of reactive oxygen species (ROS) such as superoxide, peroxyl, alkoxyl, hydroxyl, and nitric oxide [62]. The role of ROS in cells is ambiguous. On one hand, ROS are involved in cellular processes such as intercellular signalling and regulation of cell growth; however, on the other hand, they can cause damage to the cells by attacking the cell membrane, proteins, and DNA by inducing oxidative damage. Plant flavonoids are secondary metabolites important to counteract oxidative damage [63]. Under stressful conditions, flavonoids accumulate to protect the plant [64]. Due to agroinfiltration, a significant increased accumulation of flavonoids was observed for B and GS3 (Figure 3), which is a typical response to exogenous stresses in plants. However, comparing flavonoid accumulation among leaves treated with the GS3 phyto-courier, Q or B had no statistical significance on flavonoid accumulation (ANOVA one-way: control conditions: *p*-value = 0.278; agroinfiltrated: *p*-value = 0.81). Note that the antioxidant capacity was also not significantly altered by the GS3 phyto-courier or B and Q control solutions (Figure 3d). However, in the absence of biotic stress a trend was observed, which indicated a higher antioxidant capacity in leaves primed with GS3 and Q. This slight difference among treatments was, however, outweighed in the presence of the biotic stressor (Figure 3d). 

Quercetin is a particularly interesting subclass of flavonoids, since it is involved in many physiological processes such as seed germination and pollen growth, but it also has a major role in biotic and abiotic stress tolerance [14]. The GS3 formulation alone had a higher antioxidant capacity compared to the Q solution (Figure 4), which demonstrates its potential in preventing oxidative stress and protecting the plant in the presence of a stressor. Furthermore, when assessing the UV spectral signature of the quercetin freshly extracted from the GS3 phyto-courier, defined bands with A_max_ around 250 nm and 380 nm were observed. The UV spectrum of undiluted samples of the flavonoid extracted from the phyto-courier (Figure 4b) showed a sharper peak at 250 nm with respect to free quercetin (Figure 4b,c). Additionally, the spectral signature of free quercetin showed the appearance of a shoulder at ca. 330 nm which is not present in the quercetin extracted from sshLNPs. The identity of this peak cannot be here inferred; however, the appearance of absorption bands with A_max_ of 336–342 nm, which is indicative of both oxidation and formation of degradation products, was detected in quercetin samples incubated with myeloperoxidase [65].

Quercetin undergoes various chemical changes such as oxidation and the formation of quercetin quinones, whereby its stability is highly influenced by pH, temperature, physicochemical properties of the solution, as well as storage conditions and time [66]. During oxidation of quercetin, the UV spectrum progressively shifts towards lower wavelengths, and the intensity decreases [67]. Quercetin-loaded sshLNPs protect against oxidation and, in turn, preserve, over a longer period, its quality compared to a solution of free quercetin. This finding has relevance for applications of the phyto-courier under field conditions, since the flavonoid bound to hydrolysable sshLNPs possibly lengthens its stability as compared to free quercetin and thus constitutes a superior biostimulant by releasing a compound that can offer high antioxidant capacity to stressed plant cells. 

In a previous study, the external supply of quercetin in the growth medium could protect thale cress, tobacco, and duckweed against the harmful effects of reactive oxygen species (ROS) [15]. Plants treated with the phyto-courier functionalised with 2.5 mg and 25 mg of quercetin showed decreased symptoms under salt stress, as manifested by the decreased expression of genes related to stress [12] and by proteomics [13]. 

## 4. Materials and Methods

### 4.1. Experimental Set-Up and Infiltration

Suspensions of the sshLNPs functionalised with 25 mg of quercetin (GS3 formulation) and respective controls (buffer alone, B, and free quercetin, Q) [13] were prepared prior to the experiment. Polyvinyl alcohol (PVA) bags of GS3 or Q were accurately weighed and solubilised according to their weight in the appropriate volume of dispersant, to normalise the content. The dispersant was a mixture of hypromellose 2910/Pluronic L-61 (average Mn~2000) and phosphate-buffered saline (PBS) (1:1 *v*/*v*). To solubilise the content, 40 min of sonication at RT was performed. For the control B, an empty bag was solubilised in the same buffer and sonicated. The composition of the GS3 phytocourier has been previously reported [13] and is indicated in Table 1.

*N. benthamiana* seeds were first sown in a pot filled with a mixture of potting soil and sand (1:1), and, later, one-week-old seedlings were separated into individual pots. Plants were cultivated during the whole experiment in a controlled growth chamber (Fitotron, Weiss Technik, Reiskirchen, Germany) at 22.5 °C (day) and 17.5 °C (night) with a 16 h light/8 h dark photoperiod, keeping the relative humidity at 60%. Plants were four weeks when the formulations were injected into the abaxial side of the leaf lamina (two leaves per plant were infiltrated) using a needleless 1 mL syringe. The cryoconserved *A. tumefaciens* strain GV3101 was restreaked on LB agar with rifampicin 5 µg/mL and gentamycin 30 µg/mL and grown at 30 °C for two days. Thereafter, one single colony was picked and inoculated in 50 mL of liquid LB containing the same antibiotics. After two days at 30 °C and 200 rpm, the bacteria were pelleted (2500 g for 10 min), resuspended in infiltration buffer (10 mM MES, 10 mM MgCl_2_, pH 5.6) supplemented with acetosyringone at a final concentration of 150 µg/mL. The resuspended agrobacteria were brought to a final OD of 1 (at 600 nm) and kept in the dark for one hour prior to infiltration. The infiltration of agrobacteria in the leaves using a needleless syringe was carried out three days after injecting the formulations into the same leaves (conditions hereafter referred to as B agro, GS3 agro, and Q agro). As controls, infiltration buffer containing acetosyringone without *Agrobacterium* was infiltrated (conditions hereafter referred to as B, Q, and GS3). Each treatment was performed on four biological replicates. Leaves were sampled four days later, snap-frozen in liquid nitrogen, and kept at −80 °C till further use. It must be noted that more force was needed to inject GS3 and Q compared to B. Leaf tissues were partly necrotic because of the mechanical stress applied with the syringe, and therefore the concerned parts were removed immediately prior to sampling the leaves for gene expression analysis to avoid any bias caused by the necrotic tissue. 

Gene expression analysis was also performed on leaves that were primed via spraying of freshly prepared formulations: fully expanded tobacco leaves (4 weeks old) were sprayed twice (with three days’ interval between each application) prior to agroinfiltration. Leaves were sampled four days later, as described above.

### 4.2. Sampling and RNA Extraction

Infiltrated leaves were sampled and ground to a fine powder in liquid nitrogen using a mortar and a pestle. RNA was extracted with the RNeasy Mini Kit^®^ (QIAGEN, Leusden, The Netherlands) after homogenisation of cell and tissue lysates with the QIAshredder Kit^®^ (QIAGEN), following the manufacturer’s instructions. An on-column DNAse I digestion step was added to avoid DNA contamination. RNA was finally eluted using 27 µL RNAse-free water.

### 4.3. RNA Quality Check and Quantification

RNA concentrations were determined with a NanoPhotometer^®^ NP80 (Implen, Munich, Germany). Samples with A260/230 < 2 were cleaned up by RNA precipitation with ammonium acetate (NH_4_OAc) and a subsequent wash in ethanol, as previously described [68]. Thereafter, RNA was precipitated with 1/10 volume of NH_4_OAc in 2.5 volumes of 100% (*v*/*v*) cold ethanol and incubated overnight at −20 °C. RNA was recovered by centrifugation at 12,000× *g* for 20 min at 4 °C, washed with 75% (*v*/*v*) cold ethanol, centrifuged 5 min at 12,000× *g*, dried, and finally resuspended in 27 µL RNAse-free water. The RNA Integrity Number (RIN) was evaluated by capillary gel electrophoresis using a 2100 Bioanalyzer (Agilent, Santa Clara, CA, USA), according to the manufacturer’s instructions. All RINs were above 7.

### 4.4. Primer Design

Genes of interest were selected from a previous study [34]. Corresponding primer pairs were designed with Primer3Plus considering qPCR parameters [69] and verified with the OligoAnalyzer tool from Integrated DNA Technologies (https://eu.idtdna.com/calc/analyzer accessed on 12 March 2021). Primer efficiencies were determined by RT-qPCR using serial dilutions (12.5, 2.5, 0.5, 0.1, 0.02, 0.004 ng/µL) of cDNA obtained from a pool of all the samples investigated using the ProtoScript II reverse transcriptase (New England Biolabs, Leiden, The Netherlands). R^2^ and amplification efficiencies (whereby 100% amplification equals 2) were calculated using QuantStudio™ Design & Analysis Software v1.5.1 after exclusion of outliers. Primer pairs with high linearity and amplification efficiency (between 87 and 104%) were retained for further RT-qPCR analysis (Table 2).

### 4.5. RT-qPCR 

Total RNA (1 µg) was retrotranscribed to cDNA using 0.5 µL of 1.5 mM solution of random primers (Invitrogen), 1 µL of a dNTP mix (10 mM), and RTase (ProtoScript) from NEB (Leiden, The Netherlands), following the manufacturer’s instructions. For RT-qPCR, cDNA was diluted to 2 ng/µL. RT-qPCR analysis was carried out with the SYBRgreen^®^ Master mix (Takyon, Eurogentec, Liege, Belgium) in 384-well reaction plates using 10 µL reaction volume. Plates were filled using an automated dispensing device (epMotion 5073x, Eppendorf, Hambourg, Germany) for optimal reproducibility, with three technical replicates per sample. RT-qPCR runs were performed with Quantstudio 5 Real-time qPCR (Applied biosystems, Foster City, CA, USA). A melt curve analysis was performed at the end of the PCR cycles to check the presence of a single peak denoting specific amplification. 

Gene expression was determined using qBase^PLUS^ software (Biogazelle, Ghent, Belgium) [71] with the implemented geNORM tool. Data were obtained on four biological replicates. The reference genes *F-Box* and *SAND* were chosen for normalisation in the experiment where priming occurred via infiltration, while *GBP* and *UK* for the experiment where priming was carried out via spraying. These gene couples were identified as the most stable and as sufficient for data normalisation by geNORM. 

### 4.6. Preparation of Samples for Optical Microscopy

Tobacco leaves (portions devoid of the central veins) were plunged into fixation solution (glutaraldehyde/paraformaldehyde/caffeine 1%/2%/1% *v/v* in Milli-Q water). For optimal fixation, vacuum was applied to the samples for 10 min and kept at 4 °C overnight. The solution was thereafter replaced by 70% ethanol (*v*/*v*), and samples were kept at 4 °C till further use. 

To dehydrate the tissue samples, the ethanol concentration was stepwise increased (95% for 30 min, 95 *v/v* % for 1 h, 100% for 30 min, twice 100% for 1 h), and then they were transferred to a 1:1 solution of 100% (*v*/*v*) ethanol and impregnation medium (resin-Technovit 7100-Kulzer Technik Wehrheim Germany, PEG 400 2% *v*/*v*, dimethacrylate ethylene glycol 0.4% *w*/*v*) for 2 h. Subsequently, samples were transferred to 100% impregnation medium for 24 h at 4 °C. Finally, samples were included, and moulds were dried at 37 °C until complete hardening. Ten µm sections were cut with a microtome (Leica Biosystems, Nussloch, Germany) and observed under the microscope (Olympus BX51, Tokyo, Japan). 

### 4.7. Preparation of Methanolic Extracts and Quercetin Extraction from the GS3 Phyto-Courier and Q Solution

Approximately 50 mg of ground plant material were extracted with 1 mL 80% (*v*/*v*) methanol for 24 h at 40 °C under constant shaking. Samples were cooled down to room temperature, and centrifuged for 15 min at 4500 rpm [72]. Thereafter, the supernatant was transferred to a fresh tube and used for ferric reducing–antioxidant power assay (FRAP, described below), as well as UV spectrum assessment.

Furthermore, quercetin was extracted from 100 µL of GS3 phyto-courier solution and Q solution with 300 µL of 80% methanol shaking for 1 h at room temperature. Samples were centrifuged (10,000 rpm for 10 min), and the supernatant recovered. 

### 4.8. Spectrophotometric Measurements 

The FRAP assay is based on the measurement of the ability of a substance/plant extract to reduce Fe(III) to Fe(II), which forms an intense navy-blue-coloured ferric ion-TPTZ (2,4,6-tri(2-pyridyl)-1,3,5-triazine) complex. The amount of iron reduced is directly correlated to the amount of antioxidants present in the substance/plant extract and therefore its reducing capacity. The FRAP reagent was composed of acetate buffer (300 mM, pH 3.6), TPTZ (10 mM in 40 mM HCl), and FeCl_3_ (20 mM) (10:1:1 *v*/*v*) and was freshly prepared prior to the assay. For the assay, 10 µL of plant extracts from infiltrated tobacco leaves was pipetted in duplicates into a clear 96-well plate (Greiner, Kremsmünster, Austria), and 190 µL of FRAP reagent was added to each well. The assay plate was incubated for 20 min in the dark, and the absorbance was measured at 595 nm (Spark^®^ Microplate reader, TECAN, Männedorf, Switzerland). A standard curve of 0–1 mM FeSO_4_ was used to calculate the antioxidant power of the extracts. Additionally, the antioxidant capacity of GS3 and Q solutions was determined using 10 µL of each formulation for the assay. A UV spectrum was measured to qualitatively assess the quercetin present in both solutions and in a second approach to gain information about the relative abundance of flavonoids in the metabolite extracts of tobacco leaves. One hundred fifty µL of extracted quercetin from the GS3 phyto-courier or 150 µL of the extracts were pipetted into a UV-Star 96-well plate (Greiner), and the UV spectrum was measured (200 nm to 500 nm) (Spark^®^ Microplate reader, TECAN).

### 4.9. Data Analysis

Statistical analysis was carried out with IBM SPSS statistics v26 (IBM SPSS, Chicago, IL, USA). Normality and homogeneity were assessed using a Shapiro–Wilk and Levene’s test, respectively. Univariate analysis with a Tukey’s post hoc test was used when a parametric test was possible, while a nonparametric test for independent samples with a Kruskal–Wallis and Dunn’s post hoc test was used when parametric test conditions were not met.

## 5. Conclusions

The use of an experimental set-up consisting of two application modalities, one quite far from the reality of the field use (priming by infiltration) and one closer (spraying) allowed to observe three noteworthy elements hereafter resumed: (1) leaves primed with the quercetin-loaded sshLNPs showed lower expression of stress-related genes than the counterparts treated with B and Q solutions, (2) GS3 preserved the mean area of the chloroplasts under stress and prevented swelling, and (3) the sshLNPs possibly protected quercetin against oxidative degradation and could thus preserve the molecule and its antioxidant capacity for a longer time compared to a solution of free quercetin prepared at the same time and stored under the same conditions. The protective effects of the GS3 formulation are linked to both quercetin and sshLNPs, as previously proven in tomato and hemp models [12,13]. The phytocourier is indeed acting as a biostimulant and plant protectant, as it provides both an antioxidant and a beneficial nonessential metalloid. Future studies should investigate application modes of the phyto-courier that are closer to the field reality (root amendment) and under biotic conditions that are relevant to the agricultural context (by selecting specific fungal/bacterial pathogens). These studies should comprise several crop species of agricultural relevance.

## Figures and Tables

**Table 1:** Detailed composition of the formulation infiltrated into tobacco leaves.

Samples	sshLNPs			Trehalose	Quercetin	Hypromellose + Pluronic L61 Vehicle	Total Volume (25 mL PBS Addition)
	Si-NPs	Hydro-PC	Arginine: Glycine				
B						25 mL	50 mL
GS3	4 mg	16 mg	4 mg: 2 mg	4 mg	25 mg	25 mL	50 mL
Q					25 mg	25 mL	50 mL

**Table 2:** List of primers used in this study. The identifier refers to the assigned library number for genes of interest given in [34,70], as well as gene accession number for the reference genes.

Primer Name	Gene Name	Identifier	Primer Sequence fwd (5′→3′)	Primer Sequence rev (5′→3′)	Size (bp)	Efficiency %
***Genes of interest*** [34]						
*NB_Chit6*	Chitinase 6	k58:171433	GATCGCTGCTTTCTTTGCTC	CGCCTGAAGGACCATTTATC	80	95.69
*NB_Chn-A*	Endochitinase A	k72:280831	AACCTTCTTGCCACGATGTC	GTGATGACACCAAATCCAG	91	92.96
*NB_GEβGluc*	Glucan endo-1,3-beta-glucosidase	k60:395851	GCTGCTTGTTGGGAGAAAAC	AGCCTGGACCTATTGAAACC	102	95.17
*NB_PDRP1*	Pleiotropic drug resistance protein	k80:119278	AGGTTTCATCGTTCCACGAC	AGGTCTCCGAATTGAGATGC	111	92.55
*NB_PR-1a*	Pathogenesis-related protein 1A	k58:459599	TCCAACACGAACCGAGTTAC	TTGAGATGTGGGTCGATGAG	116	81.84
*NB_PR-Q*	Acidic endochitinase Q	k78:23474	CCCCAGGAGCAACATTTAAC	AATGACGCAGTGGAAGATCG	70	91.93
*NB_RNAP-β*	DNA-directed-RNA polymerase subunit beta’	k58:179615	TCCTCTTATCCCAATCTGGTG	TTGACGGACACAAACTCTGC	95	88.01
*NB_VAS*	Lipid transfer-like protein	k64:395173	TAGTCACGGTGGCGATTATG	GCGGTTTGGTGGAATTAAGG	105	91.8
*NB_WIN2*	Wound-induced protein	k58:223872	CCGTCAAAGGGTAAACATGG	GATGGAAGAGGGAATCAACG	144	97.23
*NB_OEE1*	Oxygen-evolving enhancer protein 1	k80:117138	CCACATCATTCACGGTCAAG	TGCCATCAGAAGACACTTCG	135	92.6
*NB_RuBisCO-BP*	RuBisCO large subunit-binding protein subunit beta	k64:394932	TACTGGCTTTTCCGTTCACC	TTGAGCAAGAAGCACTAGC	103	88.00
*NB_SerThrKin*	Serine/threonine-protein kinase	k78:183558	TACCGATACCGTCCAATTCC	TGCACAGTCATGGTCTTG	129	92.94
*NB_PSI-N*	Photosystem I reaction centre subunit N	k76:226809	GGCAGCAATGAACTCAAGTG	TGATTGGGAAGCCATAGAGG	100	87.00
***Reference genes*** [70]						
*Nb_F-Box*	F-box protein	Niben.v0.3.Ctg24993647 (*At5g15710*)	GGCACTCACAAACGTCTATTTC	ACCTGGGAGGCATCCTGCTTAT	127	100.8
*Nb_SAND*	Sand family protein	Niben.v0.3.Ctg25188435 (*At2g28390*)	ACCACCAACACCTATGAATGCT	CAGTCTCGCCTCATCTGGGTCA	83	88.27
*Nb_L23*	60S ribosomal protein	TC19271 (*At2g39460*)	AAGGATGCCGTGAAGAAGATGT	GCATCGTAGTCAGGAGTCAACC	110	91.46
*Nb_UK*	Uridylate kinase	EH363935 (*At5g26667*)	CTAGGAGTATATTGGAAGAGCG	AAAGATACATCGCCTTGCTGAA	107	96.72
*Nb_GBP*	GTP-binding protein	TC20872 (*At5g59840*)	GGAACTGGATTCGCAACATAGA	GACCCTTGGAAGTTGGCACAGC	114	92.5
*Nb_RdR6*	Putative RNA-dependent RNA polymerase *SDE1*	AY722008 (*At3g49500*)	TTCAGGAATGTCTTCGAGCG	AGTGATCTAGCAACCCAATGAG	134	93.3

## Data Availability

All the data presented are made available in this study.

## Supplementary Materials

The following supporting information can be downloaded at: https://www.mdpi.com/article/10.3390/ijms241814153/s1.

## References

[1] Santini A., Ghelardini L. (2015). Plant Pathogen Evolution and Climate Change. CAB Rev..

[2] Elad Y., Pertot I. (2014). Climate Change Impacts on Plant Pathogens and Plant Diseases. J. Crop Improv..

[3] Heil M., Bostock R.M. (2002). Induced Systemic Resistance (ISR) against Pathogens in the Context of Induced Plant Defences. Ann. Bot..

[4] Swarbrick P.J., Schulze-Lefert P., Scholes J.D. (2006). Metabolic Consequences of Susceptibility and Resistance (Race-Specific and Broad-Spectrum) in Barley Leaves Challenged with Powdery Mildew. Plant Cell Environ..

[5] Savary S., Ficke A., Hollier C. (2012). Crop Losses Due to Diseases and Their Implications for Global Food Production Losses and Food Security. Food Secur..

[6] Velásquez A.C., Castroverde C.D.M., He S.Y. (2018). Plant–Pathogen Warfare under Changing Climate Conditions. Curr. Biol..

[7] Ben Rejeb I., Pastor V., Mauch-Mani B. (2014). Plant Responses to Simultaneous Biotic and Abiotic Stress: Molecular Mechanisms. Plants.

[8] Pulizzi F. (2019). Nano in the Future of Crops. Nat. Nanotechnol..

[9] Shahcheraghi N., Golchin H., Sadri Z., Tabari Y., Borhanifar F., Makani S. (2022). Nano-Biotechnology, an Applicable Approach for Sustainable Future. 3 Biotech.

[10] Fraceto L.F., Grillo R., de Medeiros G.A., Scognamiglio V., Rea G., Bartolucci C. (2016). Nanotechnology in Agriculture: Which Innovation Potential Does It Have?. Front. Environ. Sci..

[11] Machado T.O., Beckers S.J., Fischer J., Müller B., Sayer C., De Araújo P.H.H., Landfester K., Wurm F.R. (2020). Bio-Based Lignin Nanocarriers Loaded with Fungicides as a Versatile Platform for Drug Delivery in Plants. Biomacromolecules.

[12] Guerriero G., Maria Sutera F., Torabi-Pour N., Renaut J., Hausman J.F., Berni R., Pennington H.C., Welsh M., Dehsorkhi A., Zancan L.R. (2021). Phyto-Courier, a Silicon Particle-Based Nanobiostimulant: Evidence from *Cannabis sativa* Exposed to Salinity. ACS Nano.

[13] Guerriero G., Sutera F.M., Hoffmann J., Leclercq C.C., Planchon S., Berni R., Hausman J.-F., Renaut J., Torabi-Pour N., Pennington H.C. (2023). Nanoporous Quercetin-Loaded Silicon-Stabilized Hybrid Lipid Nanoparticles Alleviate Salt Stress in Tomato Plants. ACS Appl. Nano Mater..

[14] Singh P., Arif Y., Bajguz A., Hayat S. (2021). The Role of Quercetin in Plants. Plant Physiol. Biochem..

[15] Kurepa J., Shull T.E., Smalle J.A. (2016). Quercetin Feeding Protects Plants against Oxidative Stress. F1000Research.

[16] Parvin K., Hasanuzzaman M., Borhannuddin Bhuyan M.H.M., Mohsin S.M., Fujita M. (2019). Quercetin Mediated Salt Tolerance in Tomato through the Enhancement of Plant Antioxidant Defense and Glyoxalase Systems. Plants.

[17] Jańczak-Pieniążek M., Migut D., Piechowiak T., Buczek J., Balawejder M. (2021). The Effect of Exogenous Application of Quercetin Derivative Solutions on the Course of Physiological and Biochemical Processes in Wheat Seedlings. Int. J. Mol. Sci..

[18] Migut D., Jańczak-pieniążek M., Piechowiak T., Buczek J., Balawejder M. (2021). Article Physiological Response of Maize Plants (*Zea mays* L.) to the Use of the Potassium Quercetin Derivative. Int. J. Mol. Sci..

[19] Luyckx M., Hausman J.F., Lutts S., Guerriero G. (2017). Silicon and Plants: Current Knowledge and Technological Perspectives. Front. Plant Sci..

[20] Guerriero G., Hausman J.F., Legay S. (2016). Silicon and the Plant Extracellular Matrix. Front. Plant Sci..

[21] Wang M., Gao L., Dong S., Sun Y., Shen Q., Guo S. (2017). Role of Silicon on Plant–Pathogen Interactions. Front. Plant Sci..

[22] Suriyaprabha R., Karunakaran G., Yuvakkumar R., Rajendran V., Kannan N. (2014). Foliar Application of Silica Nanoparticles on the Phytochemical Responses of Maize (*Zea mays* L.) and Its Toxicological Behavior. Synth. React. Inorg. Met.-Org. Nano-Met. Chem..

[23] Bao-shan L., Shao-qi D., Chun-hui L., Li-jun F., Shu-chun Q., Min Y. (2004). Effect of TMS (Nanostructured Silicon Dioxide on Growth of Changbai Larch Seedlings. J. For. Res..

[24] Sun D., Hussain H.I., Yi Z., Rookes J.E., Kong L., Cahill D.M. (2016). Mesoporous Silica Nanoparticles Enhance Seedling Growth and Photosynthesis in Wheat and Lupin. Chemosphere.

[25] Yuvakkumar R., Elango V., Rajendran V., Kannan N.S., Prabu P. (2011). Influence of Nanosilica Powder on the Growth of Maize Crop (*Zea mays* L.). Int. J. Green Nanotechnol..

[26] Farhangi-Abriz S., Torabian S. (2018). Nano-Silicon Alters Antioxidant Activities of Soybean Seedlings under Salt Toxicity. Protoplasma.

[27] Rastogi A., Tripathi D.K., Yadav S., Chauhan D.K., Živčák M., Ghorbanpour M., El-Sheery N.I., Brestic M. (2019). Application of Silicon Nanoparticles in Agriculture. 3 Biotech.

[28] Luyckx M., Hausman J.F., Isenborghs A., Guerriero G., Lutts S. (2021). Impact of Cadmium and Zinc on Proteins and Cell Wall-Related Gene Expression in Young Stems of Hemp (*Cannabis sativa* L.) and Influence of Exogenous Silicon. Environ. Exp. Bot..

[29] Liang Y., Si J., Römheld V. (2005). Silicon Uptake and Transport Is an Active Process in *Cucumis sativus*. New Phytol..

[30] El-Shetehy M., Moradi A., Maceroni M., Reinhardt D., Petri-Fink A., Rothen-Rutishauser B., Mauch F., Schwab F. (2021). Silica Nanoparticles Enhance Disease Resistance in *Arabidopsis* Plants. Nat. Nanotechnol..

[31] Grimberg Å., Carlsson A.S., Marttila S., Bhalerao R., Hofvander P. (2015). Transcriptional Transitions in *Nicotiana benthamiana* Leaves upon Induction of Oil Synthesis by WRINKLED1 Homologs from Diverse Species and Tissues. BMC Plant Biol..

[32] Ma L., Lukasik E., Gawehns F., Takken F.L.W. (2012). The Use of Agroinfiltration for Transient Expression of Plant Resistance and Fungal Effector Proteins in *Nicotiana Benthamiana* Leaves. Methods Mol. Biol..

[33] Zhao J., Ju M., Qian J., Zhang M., Liu T., Zhang K. (2021). A Tobacco Syringe Agroinfiltration-Based Method for a Phytohormone Transporter Activity Assay Using Endogenous Substrates. Front. Plant Sci..

[34] Bond D.M., Albert N.W., Lee R.H., Gillard G.B., Brown C.M., Hellens R.P., Macknight R.C. (2016). Infiltration-RNAseq: Transcriptome Profiling of *Agrobacterium*-Mediated Infiltration of Transcription Factors to Discover Gene Function and Expression Networks in Plants. Plant Methods.

[35] Falcone Ferreyra M.L., Rius S.P., Casati P. (2012). Flavonoids: Biosynthesis, Biological Functions, and Biotechnological Applications. Front. Plant Sci..

[36] Lois R. (1994). Accumulation of UV-Absorbing Flavonoids Induced by UV-B Radiation in *Arabidopsis thaliana* L. Planta.

[37] Buchweitz M., Kroon P.A., Rich G.T., Wilde P.J. (2016). Quercetin Solubilisation in Bile Salts: A Comparison with Sodium Dodecyl Sulphate. Food Chem..

[38] Aranega-Bou P., de la O Leyva M., Finiti I., Garcfa-Agustfn P., Gonzalez-Bosch C. (2014). Priming of Plant Resistance by Natural Compounds. Hexanoic Acid as a Model. Front. Plant Sci..

[39] Desmedt W., Vanholme B., Kyndt T., Maienfisch P., Mangelinckx S. (2021). Plant Defence Prming in the Field: A Review. Recent Highlights in the Discovery and Optimization of Crop Protection Products.

[40] Richmond K.E., Sussman M. (2003). Got Silicon? The Non-Essential Beneficial Plant Nutrient. Curr. Opin. Plant Biol..

[41] Guerriero G., Stokes I., Valle N., Hausman J., Exley C. (2020). Visualising Silicon in Plants: Histochemistry, Silica Sculptures and Elemental Imaging. Cells.

[42] Hussain A., Rizwan M., Ali Q., Ali S. (2019). Seed Priming with Silicon Nanoparticles Improved the Biomass and Yield While Reduced the Oxidative Stress and Cadmium Concentration in Wheat Grains. Environ. Sci. Pollut. Res..

[43] El-Serafy R.S., El-Sheshtawy A.-N., Atteya A.K.G., Al-Hashimi A., Abbasi A.M., Al-Ashkar I. (2021). Seed Priming with Silicon as a Potential to Increase Salt Stress Tolerance in *Lathyrus odoratus*. Plants.

[44] Hatami M., Khanizadeh P., Bovand F., Aghaee A. (2021). Silicon Nanoparticle-Mediated Seed Priming and *Pseudomonas* spp. Inoculation Augment Growth, Physiology and Antioxidant Metabolic Status in *Melissa officinalis* L. Plants. Ind. Crops Prod..

[45] Ahn I.P., Kim S., Lee Y.H., Suh S.C. (2007). Vitamin B_1_-Induced Priming Is Dependent on Hydrogen Peroxide and the *NPR1* Gene in *Arabidopsis*. Plant Physiol..

[46] Zhang S., Yang X., Sun M., Sun F., Deng S., Dong H. (2009). Riboflavin-Induced Priming for Pathogen Defense in *Arabidopsis thaliana*. J. Integr. Plant Biol..

[47] Jia Z., Zou B., Wang X., Qiu J., Ma H., Gou Z., Song S., Dong H. (2010). Quercetin-Induced H_2_O_2_ Mediates the Pathogen Resistance against *Pseudomonas syringae* Pv. Tomato DC3000 in *Arabidopsis thaliana*. Biochem. Biophys. Res. Commun..

[48] Uzma Jalil S., Mishra M., Ansari M.I. (2015). Current View on Chitinase for Plant Defence. Trends Biosci..

[49] Gutsch A., Keunen E., Guerriero G., Renaut J., Cuypers A., Hausman J.-F., Sergeant K. (2018). Long-term Cadmium Exposure Influences the Abundance of Proteins That Impact the Cell Wall Structure in *Medicago sativa* Stems. Plant Biol. J..

[50] Bowles D.J. (1990). Defense-Related Proteins in Higher Plants. Annu. Rev. Biochem..

[51] Békésiová B., Hraška Š., Libantová J., Moravčíková J., Matušíková I. (2008). Heavy-Metal Stress Induced Accumulation of Chitinase Isoforms in Plants. Mol. Biol. Rep..

[52] Ward E.R., Payne G.B., Moyer M.B., Williams S.C., Dincher S.S., Sharkey K.C., Beck J.J., Taylor H.T., Ahl-Goy P., Meins F. (1991). Differential Regulation of β-1,3-Glucanase Messenger RNAs in Response to Pathogen Infection. Plant Physiol..

[53] Mauch F., Staehelin L.A. (1989). Functional Lmplications of the Subcellular Localization of Ethylene-Lnduced Chitinase and β-1,3-Glucanase in Bean Leaves. Plant Cell.

[54] Stanford A., Bevan M., Northcote D. (1989). Differential Expression within a Family of Novel Wound-Induced Genes in Potato. Mol. Gen. Genet..

[55] Breen S., Williams S.J., Outram M., Kobe B., Solomon P.S. (2017). Emerging Insights into the Functions of Pathogenesis-Related Protein 1. Trends Plant Sci..

[56] Hiraga S., Ito H., Sasaki K., Yamakawa H., Mitsuhara I., Toshima H., Matsui H., Honma M., Ohashi Y. (2000). Wound-Induced Expression of a Tobacco Peroxidase Is Not Enhanced by Ethephon and Suppressed by Methyl Jasmonate and Coronatine. Plant Cell Physiol..

[57] Depège N., Bellafiore S., Rochaix J.-D. (2003). Role of Chloroplast Protein Kinase Stt7 in LHCII Phosphorylation and State Transition in *Chlamydomonas*. Science.

[58] Shang H., Li M., Pan X. (2023). Dynamic Regulation of the Light-Harvesting System through State Transitions in Land Plants and Green Algae. Plants.

[59] Mullineaux C.W., Emlyn-Jones D. (2005). State Transitions: An Example of Acclimation to Low-Light Stress. J. Exp. Bot..

[60] Pérez-Bueno M.L., Pineda M., Barón M. (2019). Phenotyping Plant Responses to Biotic Stress by Chlorophyll Fluorescence Imaging. Front. Plant Sci..

[61] Erickson J.L., Ziegler J., Guevara D., Abel S., Klösgen R.B., Mathur J., Rothstein S.J., Schattat M.H. (2014). Agrobacterium-Derived Cytokinin Influences Plastid Morphology and Starch Accumulation in *Nicotiana benthamiana* during Transient Assays. BMC Plant Biol..

[62] Biswas K., Adhikari S., Tarafdar A., Kumar R., Saha S., Ghosh P., Roychowdhury R., Hasanuzzaman M., Choudhury S., Srivastava A. (2020). Reactive Oxygen Species and Antioxidant Defence Systems in Plants: Role and Crosstalk Under Biotic Stress. Sustainable Agriculture in the Era of Climate Change.

[63] Pietta P.G. (2000). Flavonoids as Antioxidants. J. Nat. Prod..

[64] Gutsch A., Hendrix S., Guerriero G., Renaut J., Lutts S., Alseekh S., Fernie A.R., Hausman J.-F., Vangronsveld J., Cuypers A. (2020). Long-Term Cd Exposure Alters the Metabolite Profile in Stem Tissue of *Medicago sativa*. Cells.

[65] Momić T., Savić J., Vasić V. (2009). Oxidation of Quercetin by Myeloperoxidase. Adv. Phys. Chem..

[66] Wang W., Sun C., Mao L., Ma P., Liu F., Yang J., Gao Y. (2016). The Biological Activities, Chemical Stability, Metabolism and Delivery Systems of Quercetin: A Review. Trends Food Sci. Technol..

[67] Zhou A., Sadik O.A. (2008). Comparative Analysis of Quercetin Oxidation by Electrochemical, Enzymatic, Autoxidation, and Free Radical Generation Techniques: A Mechanistic Study. J. Agric. Food Chem..

[68] Mangeot-Peter L., Legay S., Hausman J.F., Esposito S., Guerriero G. (2016). Identification of Reference Genes for RT-QPCR Data Normalization in *Cannabis sativa* Stem Tissues. Int. J. Mol. Sci..

[69] Untergasser A., Nijveen H., Rao X., Bisseling T., Geurts R., Leunissen J.A.M. (2007). Primer3Plus, an Enhanced Web Interface to Primer3. Nucleic Acids Res..

[70] Liu D., Shi L., Han C., Yu J., Li D., Zhang Y. (2012). Validation of Reference Genes for Gene Expression Studies in Virus-Infected *Nicotiana Benthamiana* Using Quantitative Real-Time PCR. PLoS ONE.

[71] Hellemans J., Mortier G., De Paepe A., Speleman F., Vandesompele J. (2008). QBase Relative Quantification Framework and Software for Management and Automated Analysis of Real-Time Quantitative PCR Data. Genome Biol..

[72] Wong C.C., Li H.B., Cheng K.W., Chen F. (2006). A Systematic Survey of Antioxidant Activity of 30 Chinese Medicinal Plants Using the Ferric Reducing Antioxidant Power Assay. Food Chem..

