# Supplementary material for: A Study on the Use of the Phyto-Courier Technology in Tobacco Leaves Infected by Agrobacterium tumefaciens
Source: Int J Mol Sci. 2023 Sep 15;24(18):14153. doi: 10.3390/ijms241814153 (PMC10531687; doi:10.3390/ijms241814153)
Supplement: Supplementary file 1 [file ijms-24-14153-s001.zip › ijms-2598093-supplementary.pdf]

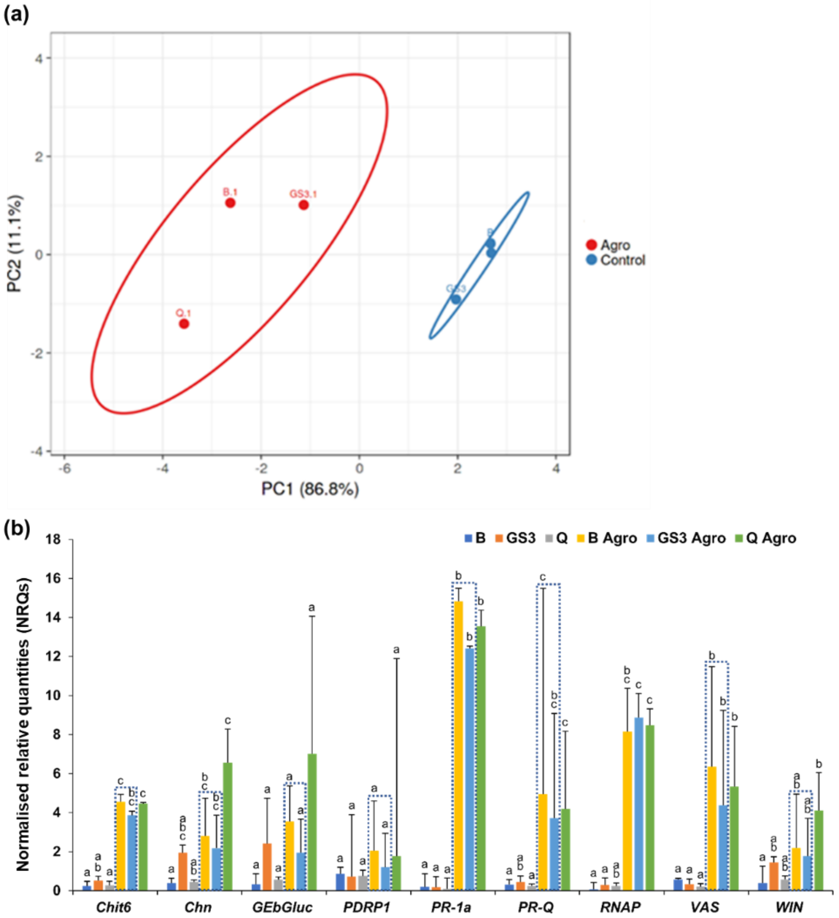

Figure S1. (a) PCA of the gene expression values (red ellipse: stressed leaves, blue ellipse: control leaves); (b) Gene expression data expressed as normalized relative quantities (NRQs). The error bars refer to the standard deviation calculated from four biological replicates. Different letters indicate statistically significant differences among groups (p-value < 0.05). An ANOVA one-way analysis followed by Tukey's test was applied for *GEbGluc* [F(5) = 2.802, p-value = 0.048] *PDRP1* [F(5) = 3.248, p-value = 0.29], *WIN* [F(5) = 3.654, p-value = 0.019], whereas a Kruskal-Wallis test with Dunn's post-hoc test was applied for *Chit6* [X^2(5) = 17.92, p-value = 0.003], *Chn* [X^2(5)= 11.29, p-value = 0.46], *PR-1a* [X^2(5) = 17.63, p-value = 0.003], *PR-Q* [X^2(5) = 17.77, p-value = 0.003], *RNAP* [X^2(5) = 18.47, p-value = 0.002], *VAS* [X^2(5) = 17.74, p-value = 0.003]. The dotted areas show the trend towards decreased expression values in the GS3-treated leaves as compared to B-treated samples. B: buffer; GS3: phyto-courier formulation containing 25 mg quercetin; Q: quercetin; Agro: agroinfiltrated.

Table S1. Statistical parameters of the data shown in Figure 1.

**Statistical parameters of Figure 1a**	**Statistical parameters of Figure 1b**
OEE1 [F(2,6) = 1.029, *p*-value = 0.413]	*OEE1* [F(2,6) = 0.330, *p*-value = 0.731]
PSI-N [F(2,6)= 18.033, *p*-value = 0.003]	*PSI-N* [F(2,6)= 0.969, *p*-value = 0.432]
RuBisCO-BP [X^2(2)= 4.622, *p*-value = 0.099]	*RuBisCO-BP* [F(2,6)=2.103, *p*-value = 0.203]
SerThrKin [F(2,6)= 0. 161, *p*-value = 0.855]	*SerThrKin* [F(2,6)= 6.679, *p*-value = 0.030]
Chit6 [F(2,6) = 5.001, *p*-value = 0.053]	*Chit6* [F(2,6) = 0.106, *p*-value = 0.856]
Chn [F(2,6) = 4.374, *p*-valu e= 0.067]	*Chn* [X^2(2)= 1.156 , *p*-value = 0.561]
GEbGluc [F(2,6) = 5.270, *p*-value = 0.048]	*GEbGluc* [F(2,6) = 2.051, *p*-value = 0.210]
PDRPI [F(2,6) = 1.694, *p*-value = 0.261]	*PDRPI* [F(2,6) = 1.021, *p*-value = 0.451]
PO [F(2,6) =1.623, *p*-value = 0.273]	*PO* [F(2,6) =18.658, *p*-value = 0.003]
PR-1a [F(2,6) = 1.576, *p*-value = 0.282]	*PR-1a* [F(2,6) = 6.573, *p*-value = 0.031]
PR-Q [F(2,6) = 11.781, *p*-value = 0.008]	*PR-Q* [X^2(2)= 0.089, *p*-value = 0.957]
RNAP [F(2,6) = 1.955, *p*-value = 0.222]	*RNAP* [F(2,6) = 19.653, *p*-value = 0.002]
VAS [F(2,6) = 7.394, *p*-value = 0.024]	*VAS* [F(2,6) = 0.974, *p*-value = 0.430]
WIN2 [F(2,6) = 0.224, *p*-value = 0.806]	*WIN2* [F(2,6) = 1.787, *p*-value = 0.246]
